# Oximes: Inhibitors of Human Recombinant Acetylcholinesterase. A Structure-Activity Relationship (SAR) Study

**DOI:** 10.3390/ijms140816882

**Published:** 2013-08-16

**Authors:** Vendula Sepsova, Jana Zdarova Karasova, Jan Korabecny, Rafael Dolezal, Filip Zemek, Brian J. Bennion, Kamil Kuca

**Affiliations:** 1 Department of Toxicology, Faculty of Military Health Sciences, University of Defence, Trebesska 1575, 500 01 Hradec Kralove, Czech Republic; E-Mails: sepsova@pmfhk.cz (V.S.); korabecny@pmfhk.cz (J.K.); zemek.filip@gmail.com (F.Z.); 2 Department of Public Health, Faculty of Military Health Sciences, University of Defence, Trebesska 1575, 500 01 Hradec Kralove, Czech Republic; E-Mail: karasova@pmfhk.cz; 3 University Hospital, Biomedicinal Research Centre, Sokolska 581, 50005 Hradec Kralove, Czech Republic; E-Mail: rafael.dolezal@fnhk.cz; 4 Biosciences and Biotechnology Division, Lawrence Livermore National Laboratory, 7000 East Ave, Livermore, CA 94550, USA; E-Mail: Bennion1@llnl.gov; 5 Center of Advances Studies, Faculty of Military Health Sciences, University of Defence, Trebesska 1575, 500 01 Hradec Kralove, Czech Republic

**Keywords:** oximes, acetylcholinesterase, inhibitors, SAR study

## Abstract

Acetylcholinesterase (AChE) reactivators were developed for the treatment of organophosphate intoxication. Standard care involves the use of anticonvulsants (e.g., diazepam), parasympatolytics (e.g., atropine) and oximes that restore AChE activity. However, oximes also bind to the active site of AChE, simultaneously acting as reversible inhibitors. The goal of the present study is to determine how oxime structure influences the inhibition of human recombinant AChE (*hr*AChE). Therefore, 24 structurally different oximes were tested and the results compared to the previous eel AChE (*Ee*AChE) experiments. Structural factors that were tested included the number of pyridinium rings, the length and structural features of the linker, and the number and position of the oxime group on the pyridinium ring.

## 1. Introduction

Acetylcholinesterase (AChE) reactivators (*i.e.*, oximes) were developed for the treatment of organophosphate intoxication. In 1955, Wilson and Ginsburg [1–3] in the United States and Childs in the United Kingdom independently prepared and presented the compound 2-pyridine aldoxime methiodide (2-PAM) as a reactivator with great potential for the treatment of organophosphorus poisoning (OP), which includes nerve agents and pesticides. Exposure to organophosphorus compounds (OPC) such as soman, sarin, or VX causes respiratory failure resulting from the paralysis of the diaphragm and intercostal muscles, depression of the brain respiratory centre, and bronchospasms. Moreover, bronchial secretions increase and death arises from suffocation [4]. OPC intoxication has become common due to their use in agriculture as pesticides and the increased threat of nerve agent misuse during military conflicts [5,6] and by terrorists [7].

The toxic effect of these OPCs arises from the inhibition of acetylcholinesterase (EC 3.1.1.7) by the formation of a covalent bond between the organophosphate and the hydroxyl group of the serine residue (203) in the enzyme’s active site. Thus, AChE is not able to hydrolyze the neurotransmitter acetylcholine (ACh) in the synaptic cleft, thereby increasing the concentration of ACh in the synapse. The standard treatment for OPC poisoning involves the administration of an anticonvulsant (e.g., diazepam) that relieves muscle fasciculation and parasympatolytics (e.g., atropine) to block the effects of overstimulated cholinergic receptors in the peripheral nervous system caused by the high ACh concentration. Oxime based reactivators (e.g., PAM-2, HI-6, obidoxime) regenerate active AChE by displacing the phosphonyl moiety from the inhibited enzyme. First, the oxime-OPC-AChE complex is created; then the enzyme is dephosphonylated and the phosphorylated reactivator presumably leaves the AChE active site. At this stage, the enzyme activity is restored. Reactivators are not able to re-establish the enzyme’s physiological function after aging (spontaneous dealkylation of the OPC side chain). The rate of reactivation is dependent on the OPC inhibitor structure, the enzyme source, the reactivator structure, the reactivator concentration, and the rate of aging [8]. Despite an enormous amount of effort to develop a universal reactivator, none are sufficiently effective against all OPC types.

Oxime based reactivators can also bind to the active site of native AChE as reversible inhibitors. It is known that they can bind to allosteric sites as well, thereby inducing an indirect protection of the AChE active site [8]. The goal of the present study is to determine how reactivator structure influences the reversible inhibition of human AChE. Therefore, we assayed 24 structurally different oxime based reactivators to establish a structure-activity relationship (SAR). We found that the bis-pyridinium linker chain length is proportional to increase reversible inhibition of the reactivator. We also compared IC_50_ values of the same reactivators against our previously published data for the *Ee*AChE enzyme. In general, the *hr*AChE is more sensitive to inhibition than the *Ee*AChE isoform. Additionally, computational docking was performed to help rationalize, at the atomic level, the low IC_50_ values and differences in IC_50_ between *hr*AChE and *Ee*AChE enzymes.

The SAR of the bis-pyridinium oxime inhibition of AChE obtained from this study will be useful in designing and subsequent synthesis of new peripherally acting reactivators. As these compounds contain quaternary ammonium moieties, they have limited permeability through the blood-brain barrier [9,10]. Furthermore, they may be used as prophylaxis against OPCs or as new drugs for the treatment of Myasthenia gravis, and in anesthetic practice to reverse the skeletal muscle relaxation induced by non-depolarizing neuromuscular blocking agents [11].

## 2. Results and Discussion

A large number of pyridinium oximes have previously been tested for their efficacy in reactivating *hr*AChE. These studies have shown which structural factors are important for activity. These factors included the number of quaternary ammonium fragments, the length and the structural features of the connecting linker between pyridinium rings, and the number and position of the oxime group in the pyridinium ring [12]. In this study, we followed up on our previous efforts to understand the correlation of important structure factors operating during reversible inhibition and in the reactivation process of the *hr*AChE enzyme. As in the previous *Ee*AChE study [13], the reactivators ability to reversibly inhibit *hr*AChE is expressed as an IC_50_ value.

### 2.1. Mono-Pyridinium Reactivators with Different Oxime Group Positions

**Figure 1:**
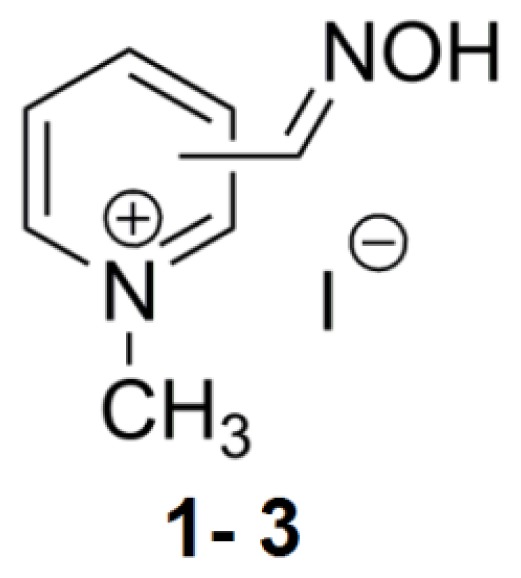
Chemical structures of assayed mono-pyridinium reactivators.

The first described reactivator, pralidoxime (2-PAM), has been shown to inhibit AChE activity [3]. This was confirmed according to our results in Table 1, displaying an IC_50_ value of 45 mM. Compound **2** with oxime group in the *meta* position shows a similar value of 41 mM (Figure 1). Interestingly, 4-PAM fails to inhibit 50% of *hr*AChE activity with the concentrations used in this study. 2-PAM is considered a more potent reactivator than 4-PAM [14], but we find that 2-PAM also has a greater inhibitory potency. However, a reactivator with two quaternary ammonium functional group atoms has been shown to have higher affinity for both the intact and OPC inhibited AChE compared to these mono-quaternary compounds [15].

### 2.2. Bis-Pyridinium Reactivators with Various Connecting Chain Lengths

**Figure 2:**
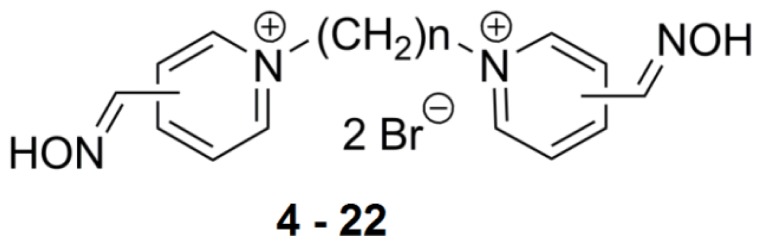
Chemical structures of assayed bis-quaternary ammonium reactivators.

The connecting link between the two quaternary ammonium moieties atoms has a significant effect on the reactivation rate and overall toxicity [16]. To observe whether the same effect was present for inhibition potency, we assayed 12 bis-pyridinium reactivators with the oxime group at the *para* position and varying linker length in the *hr*AChE enzyme (Figure 2). Compounds that are commonly shown to be AChE reactivators (1–5 carbons in connecting chains) have lower inhibitory potency. AChE inhibition increases with the length of chain as IC_50_ values decrease from 21 mM for **4** to 1 mM for **8** (Table 2). Bis-pyridinium aldoximes with linkers composed of 2–10 methylene groups were tested by Kassa *et al* [17] and it was confirmed that these compounds were not able to reactivate AChE inhibited by tabun or cyclosarin. Remarkably, based on our current results, the extended linker decreases the IC_50_ values to 55 μM for **13**. Compounds with linkers of 9–12 methylene groups possess similar IC_50_ values. This finding could be a result of the bulkier molecules which allows interaction with more amino acid residues occupying AChE active site. The distance of 10 carbons appears to be ideal in connecting the peripheral and catalytic sites of AChE [18]. Thus, bis-pyridinium reactivators can occupy both sites (peripheral aromatic site and catalytic anionic site) and confer the compounds inhibitory ability towards AChE as confirmed in our study. The connecting linker does not play a direct role in the dephosphonylation process; however, it is important in distribution, elimination, and AChE reactivation rates (e.g., in the binding mechanism) [19].

### 2.3. Bis-Pyridinium Reactivators with 3 or 4 Carbon Connecting Linker and Various Positions of the Oxime Group on the Pyridinium Ring

Tri or tetra-carbon linkers appear to be the most suitable for the reactivation process of soman, tabun, or cyclosarin-inhibited AChE [19] (Figure 2, *n* = 3,4). These derivatives of trimedoxime (compound **6**) were prepared by Musilek *et al.* and superior reactivation ability was confirmed for them over trimedoxime [20]. Interestingly, the IC_50_ for analogue **6** measured in *hr*AChE is 10^−2^ M while other reactivators **16**–**20** showed values in the mM range (10^−3^ M) (Table 2). The highlighted inhibitor in this series was compound **16** with the oxime group in *ortho* position (2.2 mM). Similar results were observed for reactivators connected with 4 methylenes (**7**, **21** and **22)** with IC_50_ values also in the mM range. Overall, the results point to compound **21** (1.1 mM) with the oxime group in the *ortho* position.

### 2.4. Bis-Pyridinium AChE Reactivators—Substitutions and Double Bond in Linker

**Figure 3:**
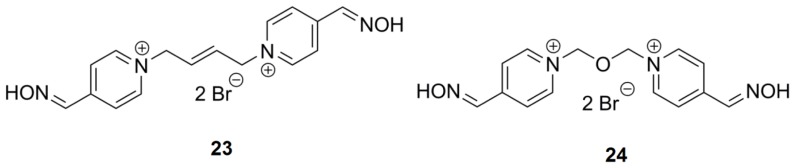
Chemical structures of the bis-pyridinium reactivators with an oxygen substitution or double bond in the connecting linker.

The insertion of a double bond increases the reactivation ability, however toxicity is also increased [20]. Incorporating an oxygen atom instead of a double bond may reduce toxicity. The IC_50_ values are similar for **7** and **24**, 3 × 10^−3^ M and 8 × 10^−3^ M, respectively. As a double bond is slightly shorter in length, the linker with a double bond (Figure 3) is between 3 and 4 carbons and the IC_50_ values are reflective of this structure (Table 3). In addition, a double bond does not permit rotation and therefore, passage of the reactivators with this moiety through the narrow AChE cavity may be more complicated. Substitution of oxygen in the connecting chain does not have a big impact on *hr*AChE inhibition in comparison to other compounds with similar linker size in this study.

### 2.5. SAR for Reversible Inhibition of hrAChE

**Figure 4:**
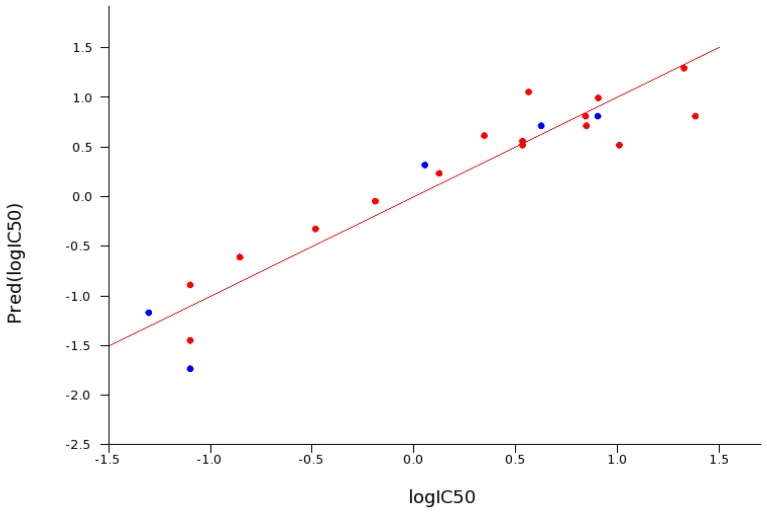
Experimental *versus* predicted pIC_50_ values for the 21 compounds in this study. The IC_50_ prediction was based on the eight descriptors identified by principle component analysis. The “leave one out”—Q^2^, correlation was 0.86. Red dots are compounds used in the training set and blue are from the test set. In general, extension of the linker length increased the inhibition potency of the bis-pyridinium oximes. Compounds **4**, **6**, **24** are currently approved as organophosphorus poisoning (OP) antidotes in various parts of the world.

The inhibition data for 21 bis-pyridinium compounds were used to build a statistical model of inhibitory potential. A principle component regression was performed to determine a SAR for inhibition (descriptors are detailed in the Experimental Section). By far, the largest determinant from this particular set of compounds is the length (>5 carbon atoms) as seen by the contribution of rotatable bonds, molecular weight and molar refractivity of the linker between pyridinium rings. The second major contributor to inhibition potency is the position of the oxime groups on the rings (*ortho* derivatives resulted in highest anti-AChE activity) (Figure 4). More potent compounds were predicted from these calculations and will be assessed in future work. As several compounds in the data set are currently approved reactivators (they contain one to three carbon linkers), a drastic reduction in activity can be found among them (IC_50_ ~10^−3^ M) suggesting a possible negative correlation with reactivation.

### 2.6. Reversible Inhibition of *Ee*AChE *versus hr*AChE

**Figure 5:**
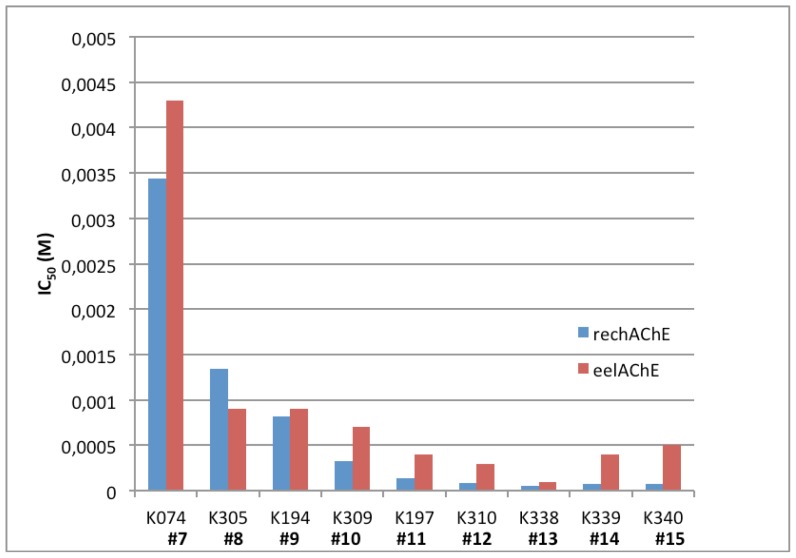
Comparison of various IC_50_ values of bis-pyridinium compounds in *hr*AChE and *Ee*AChE. In general, the *hr*AChE enzyme is more easily inhibited by oxime reactivators than the *Ee*AChE enzyme.

A set of 24 reactivators were assayed for reversible inhibition of *Ee*AChE by Sepsova *et al.* [13]. The SAR results are similar to those for *hr*AChE as presented in this study. In general, the IC_50_ values reported in this study for *hr*AChE are equal to or lower than those reported for the eel enzyme. In the group of bis-pyridinium reactivators with variable length linkers, compound **13** was the strongest inhibitor of *hr*AChE with an IC_50_ of 55 μM, which is 50% lower than in the *Ee*AChE enzyme (100 μM). Compounds **4**–**6**, **10**–**15**, **17**, **18**, **20** have IC_50_ values ranging between two and 40-fold lower for *hr*AChE in comparison to *Ee*AChE (Table 2 and Figure 5). The seven remaining compounds have equal or higher IC_50_ values *in hrAChE* in comparison to *EeAChE*. Unfortunately, there is not enough information to determine a cause for the species differences in these IC_50_ values. However, based on the inhibition data, the native *hr*AChE enzyme is more sensitive to inhibition by these oxime containing reactivators. For the most potent inhibitors, therapeutically achievable concentrations equivalent to IC_50_ values of 10^−4^ and lower are possible [10]. This means that general extrapolation of non-human AChE data needs to account for possible species differences. Although these data are determined from native, non-OPC adducted enzymes, they suggest that reversible inhibition should be included in assays for new lead compounds with reactivation potential.

### 2.7. Molecular Docking

Molecular docking studies were carried out on compound **13** in order to rationalize its plausible interactions within the active sites of *hr*AChE, *Ee*AChE and *Torpedo califonica* AChE (*Tc*AChE). The crystal structures of *Tc*AChE with bis(7)-tacrine (PDB ID: 2CKM) and *Ee*AChE (PDB ID: 1C2O) were chosen because of an assumption of a comparable binding mode for **13**/*hrAChE* complex (for *hr*AChE complexed with fasciculin-2: PDB ID: 1B41).

**Figure 6:**
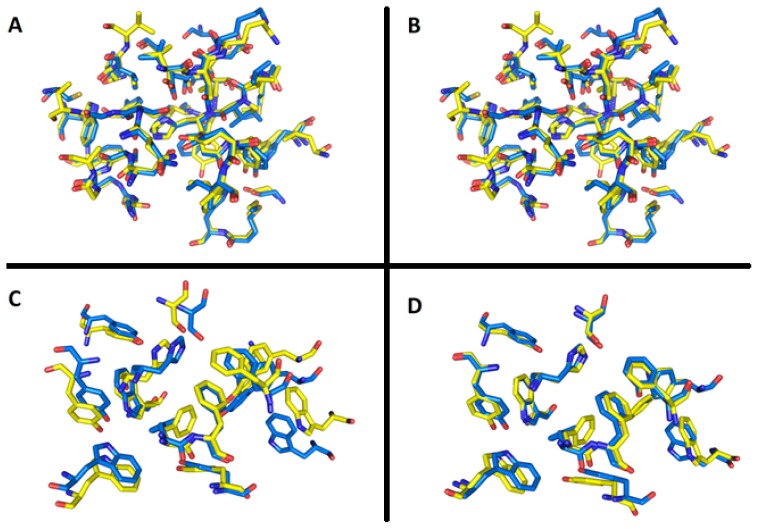
Superimposition of *Tc*AChE active site (yellow) on *Ee*AChE active site (blue) residues within R = 8 Å spherical selection calculated by super (**A**) root-mean square deviations (RMSD) (super) = 0.400 Å and align (**B)** RMSD (align) = 0.416 Å algorithm. Alignment of 13 flexible residues is rendered in the same manner (**C**) RMSD (super) = 0.586 Å; (**D**) RMSD (align) = 0.536 Å.

**Figure 7:**
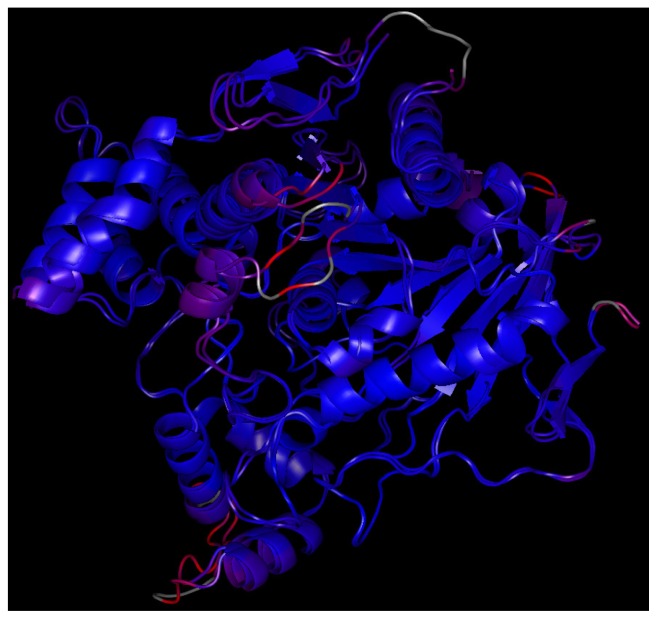
Overall superimposition of *Tc*AChE on *Ee*AChE colored by C_α_ RMSD (“super” algorithm used, 449 C_α_ carbon atoms matched). Dark blue is a good alignment; higher deviations are in orange/yellow/red. Residues omitted in the alignment are white. Minimum C_α_ distance: 0.04 Å, maximum C_α_ distance: 5.25 Å, average C_α_ distance: 0.71 Å, C_α_ RMSD = 0.545 Å.

Selection of *Tc*AChE for docking simulation deserves further comment. *In vitro* assessment was carried out with *Ee*AChE. However, this enzyme is available from the Protein Data Bank (PDB.org) only in very low resolution [21]. To the best of our knowledge, amino acid residues within both *Tc*AChE and *Ee*AChE active sites are conserved and the sequence corresponds one to another preserving important amino acid residues (e.g., for peripheral anionic site: Trp286 in *Ee*AChE = Trp279 in *Tc*AChE; for the active site: Trp86 in EeAChE = Trp84 in *Tc*AChE; the catalytic triad is maintained and residues in mid-gorge are conserved). In order to decide how much the enzymes *Tc*AChE and *Ee*AChE are similar and/or different in quantitative terms, we performed a similarity study of the X-ray structural enzyme models prior to docking simulation. Using two aligning functions—“super” and “align”—available in Pymol viewer, root-mean square deviations (RMSD) of the active sites as well as of the whole enzyme structures were calculated. To approximate the enzyme active sites, residues included in a sphere of *R* = 8 Å, around His440 in the case of *Tc*AChE and around His447 in the case of *Ee*AChE, were selected. The radius was chosen empirically to encompass the key part of the enzyme active sites. Superimposition of the active site *Tc*AChE model (mobile part) to the active site *Ee*AChE model (stationary part) by the “super” algorithm provided optimal overlap with RMSD (super) = 0.400 Å. Matching residue pairs within the active sites, whose distance was minimized during the RMSD calculations, are listed in Table 4. Applying the “align” algorithm (Pymol) similar results were obtained: RMSD (align) = 0.414 Å (Figure 6). Alignments restricted to 13 residues set as flexible in docking simulations (see below) yielded RMSD (super) = 0.586 Å and RMSD (align) = 0.536 Å (Figure 6). Depending on the algorithm used, superimposing of the whole enzymes converged to these values: RMSD (super) = 0.616 Å (3056 atom pairs matched), RMSD (align) = 0.624 Å (3078 atoms pairs matched). Considering only alpha carbons, even lower RMSD values were achieved: C_α_ RMSD(super) = 0.545 Å, C_α_ RMSD(align) = 0.550 Å (Figure 7). In all RMSD calculations, the structures of both enzyme models were treated as rigid.

According to the results obtained via RMSD calculations, we hypothesized significant similarity of *Tc*AChE and *Ee*AChE enzymes. Afterwards, this assumption was re-evaluated in docking simulation rendered in complexes **13**/*Tc*AChE and **13**/*Ee*AChE.

**Figure 8:**
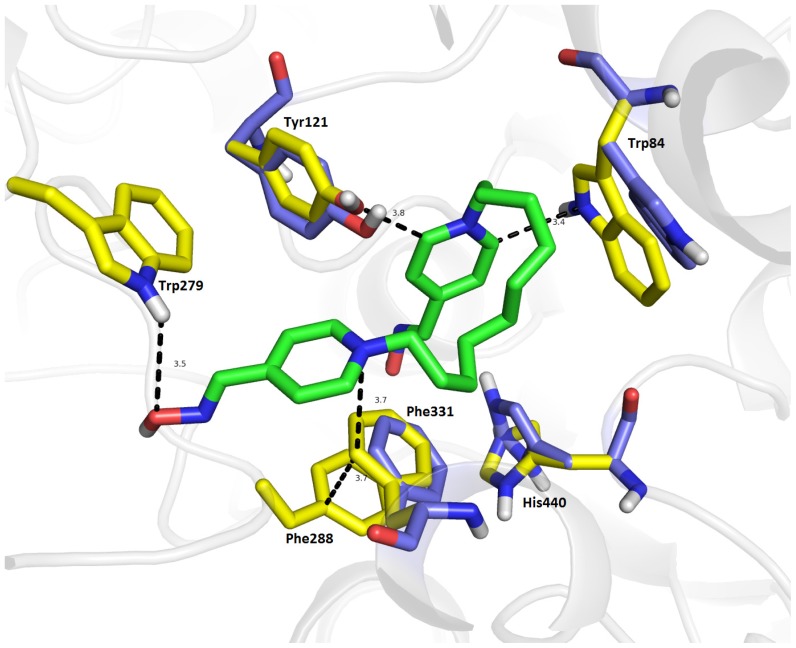
The top-scoring pose for compound **13** (green) in in the active site of *Tc*AChE. The original spatial conformation of amino acid residues are rendered in blue, amino acid residues in computationally determined alternate conformations are shown in yellow, and the rest of the enzyme is displayed in a cartoon representation. For the sake of clarity, original conformation of Trp279 is not depicted since no structural modification was observed.

Considering **13**/*Tc*AChE complex, docking calculations were carried out using the *Tc*AChE crystal structure (Figure 8). The recorded binding energy for **13**/*Tc*AChE complex is −9.3 kcal/mol, which is slightly higher compared with the **13**/*hr*AChE complex (−8.7 kcal/mol) but lower than it appeared for **13**/*Ee*AChE complex (−10.1 kcal/mol). The distal pyridinium ring exerts a similar spatial conformation as in the *hr*AChE, providing a hydrogen bond between Trp279 (3.5 Å, correspond with Trp286 in *hr*AChE) and oxime function at the peripheral anionic site of *Tc*AChE. Next, the peripheral pyridinium moiety is stacked by a T-shaped cation-π interaction to Phe331 (3.7 Å), which is additionally stabilized directly by a parallel sandwiched π-π bonding to Phe288 (3.7 Å). Within the anionic site, Trp84 demonstrates a similar rearrangement as Trp86 within *hr*AChE with a rotation of ~94°. Moreover, these conformational changes of Trp84 lead to a stabilization of the proximal pyridinium ring via a cation-π interaction (3.4 Å). Supplementary π-π bonding is formed with Tyr121 in a T-shaped conformation which is slightly distorted from its original structure (~28°). The catalytic triad is more disrupted when compared to the **13**/*hr*AChE complex yielding a drastically altered conformation of His440 (~74°). This change in the residue orientation results in hydrogen-bonding with oxime group of proximal pyridinium ring (3.2 Å). Glu199 is not affected in this study, while Ser200 shows a strong hydrogen bond with an oxime group (1.7 Å). Likewise, the 10-carbon spacer bridges the gorge making aliphatic-π interactions with Phe330 (3.2 Å) and Tyr334 (4.6 Å).

**Figure 9:**
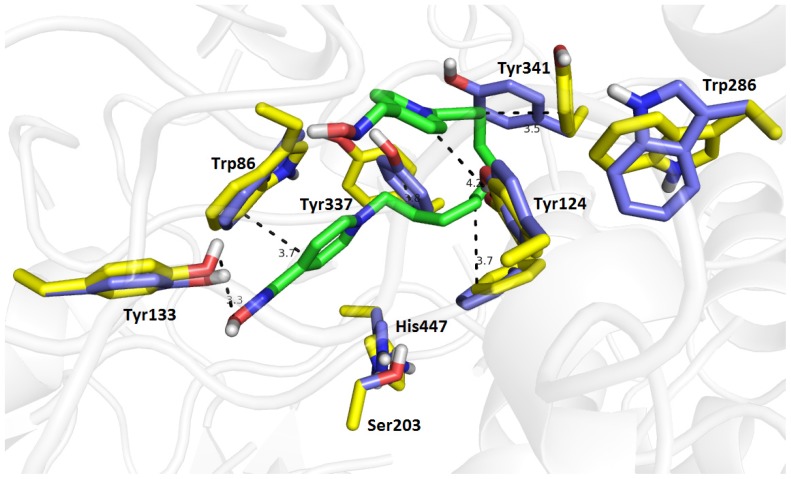
The top-scoring pose for compound **13** (green) in the active site of *Ee*AChE. The original spatial conformation of amino acid residues are rendered in blue, amino acid residues in computationally determined alternate conformations are shown in yellow, and the rest of the enzyme is displayed in a cartoon representation.

Related to *Ee*AChE, the corresponding ligand conformation of **13** exhibited larger geometrical differences that do not correlate with the assumption implied by the RMSD calculation results (Figure 9). The proximal pyridinium ring of **13** provided a parallel π-π interaction with Trp86 (3.7 Å) in the CAS of the enzyme active site. This might be additionally stabilized by the hydrogen bond between the OH group from Tyr133 and the oxime moiety (3.3 Å); the interaction was only observed for **13**/*Ee*AChE complex. The distal pyridinium ring did not approach Trp286—one of the most important aromatic residues in the PAS—and only provided a T-shaped π-π interaction with Tyr124 (4.2 Å). This might explain the decrease in binding affinity between *hr*AChE and *Ee*AChE found in *in vitro* experiments. Furthermore, several hydrophobic interactions contributed to the linker orientation in the mid-gorge of *Ee*AChE (e.g., Tyr341, Tyr337). The presence of **13** in the active site of *Ee*AChE has no impact on catalytic triad. It is important to note that these results must be taken with caution due to low resolution of the *Ee*AChE crystal structure.

**Figure 10:**
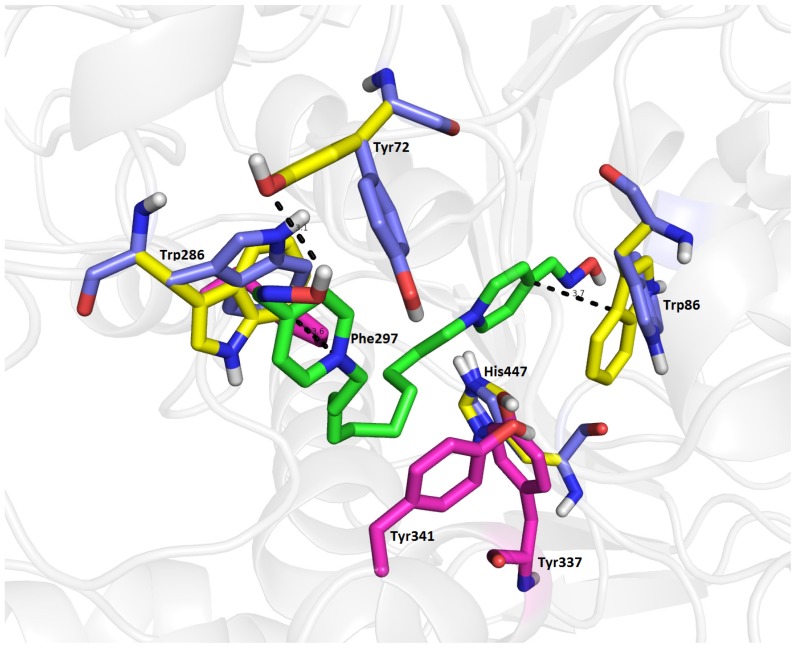
Compound **13** (green) docked into the *hr*AChE active site showing its interactions with various amino acid residues. The original spatial conformations of amino acid residues are shown in blue, amino acid residues in computationally determined alternate conformations are shown in yellow, and the rest of the enzyme is displayed in a cartoon representation. Other residues with important interactions with the oxime are shown in magenta. Significant changes are seen in several important active site residues upon oxime binding.

The results for the *hr*AChE docking calculation are shown in Figure 10. The original crystal structure positions of important amino acid residues are displayed in blue, those that were computationally reoriented in yellow, and residues having direct interactions with **13** are shown in magenta (Figure 10). The top-scored docking pose for **13**/*hr*AChE complex (−8.7 kcal/mol) shows important π-π and cation-π interactions (Figure 10). The peripheral pyridinium moiety is oriented ring-to-ring facing Trp286 (3.6 Å). For this interaction to occur, the indole skeleton of Trp286 (yellow) undergoes a ~63° rotation relative to its conformation in the original position in the *hr*AChE crystal structure (blue Trp286). Tyr72 is also dramatically reoriented (~59°) to provide hydrogen bonding with the oxime group of the distal pyridinium moiety with a distance of 3.1 Å. Interestingly, Tyr72 is excluded from parallel π-π bonding and gives only a weak π-π, T-shaped interaction (4.2 Å). The largest structural alterations, upon the binding of **13** to *hr*AChE, are observed for Trp86 in the cation-π site that bind to the distal pyridinium ring (3.7 Å). The atoms of this residue show a ~60° rotation from their original position in the *hr*AChE crystal structure. The catalytic triad is only slightly affected by **13**, a very frail π-π interaction is observed between the distal pyridinium ring and His447 (4.5 Å), which is slightly rotated. The side chain of Glu202 is close to the oxime group (3.7 Å) forming a hydrogen donating bond. In the gorge between both pyridinium units, the 10-carbon spacer spans the length of the active site gorge and is consistent with the idea of a dual binding site interaction as mentioned above. In the middle of the gorge, the aliphatic chain of **13** was surrounded by the phenyl rings of Phe297 (3.4 Å), Tyr337 (4.3 Å) and Tyr341 (3.4 Å) stabilizing **13/***hr*AChE. The distance between both moieties is approximately 20 Å which is in accordance with the distance between peripheral anionic site and catalytic site of *hr*AChE [22].

The results from the docking calculations presented in this study are in general agreement with previous docking studies for non-oxime based, bis-pyridinium-inhibitors of cholinesterases, which predicted similar orientations within active site of AChE [23,24]. The low IC_50_ values for compound **13** further suggest a characteristic dual binding site that was elucidated by *in silico* studies. Shorter pyridinium ring based reactivators (e.g., trimedoxime) showed less activity presumably because they do not reach both binding sites in the *hr*AChE gorge. The docked poses of the **13** in *hr*AChE and *Tc*AChE interact with similar residues, while **13**/*Ee*AChE complex revealed different binding pose unexpectedly to calculated RMSD for *Tc*AChE and *Ee*AChE. Although the conformational changes in the *Tc*AChE enzyme appear to be more dramatic in the catalytic triad residues, the similar binding mode in the gorge suggests that inhibition of both enzymes would be similar in magnitude.

## 3. Experimental Section

### 3.1. Chemicals

All assayed reactivators were previously synthesized at the Department of Toxicology, Faculty of Military Health Science, University of Defence, Hradec Kralove, Czech Republic [25]. Phosphate buffer, human recombinant AChE (*hr*AChE), DTNB (5,5′-dithiobis (2-nitrobenzoic) acid) and acetylthiocholine iodide were purchased from Sigma-Aldrich (Prague, Czech Republic).

### 3.2. The Measurement of IC_50_ of *hr*AChE

The activities of *hr*AChE were evaluated by the standard spectrophotometric Ellman’s method. Acetylthiocholine iodide was used as a substrate and DTNB was used as the chromogen. The standard wavelength of 412 nm was used [26,27]. The absorbance was determined using a Helios Alpha (Thermo Scientific, Loughborough, UK) spectrophotometer. The results were then analyzed by using the standard statistical software package, Prisma 4.0 (GraphPad Software, La Jolla, CA, USA).

In vitro measurements were completed as follows: A solution of *hr*AChE (90 μL, activity was previously established) was pipetted into the cuvette. Subsequently, 10 μL of the selected reactivator in concentrations from 10^−1^ to 10^−8^ M were added. This mixture was then incubated for 10 min under laboratory temperature (20 ± 2 °C). Then, 200 μL of DTNB and 600 μL of phosphate buffer (0.1 M, pH 7.4) were added. The reaction was started by adding acetylthiocholine iodide (100 μL, for 1 μM). This procedure was repeated three times for each incubation.

Reactivators in higher concentrations may split DTNB; this process is known as oximolysis and produces false-positive results [28]. To eliminate this issue, a portion of *hr*AChE was replaced by distillated water. Subsequently the same portions of other reagents were added. Acquired measurements were then deducted from the calculated *hr*AChE activity values. Actual activity of the enzyme (the blind sample) was established for all concentration series. The reactivator was replaced by water in cuvette and obtained values were calculated as 100% of the enzyme’s activity [13].

Structure activity relationships were calculated with Canvas [29] (version 1.5, Schrödinger, LLC, New York, NY, USA, 2011) from the Schrodinger software suite. Briefly, all default molecular descriptors were calculated for the 21 bis-pyridinium oxime compounds. Principle component analysis was performed to determine which features sample the most variance in the data. These descriptors are AlogP, electrotopological state, hydrogen bond acceptors, molar refractivity, molecular weight, polar surface area, and the number of rotatable bonds. These features were then used in principal component regression to build a model. The model was built with 15 compounds randomly selected from the original set of 20. The remaining 5 were then used in testing the generated model.

### 3.3. Docking Study

Docking calculations were performed using AutoDock Vina [30]. The molecular models were built and minimized with UCSF chimera 1.3 (Amber Force Filed) [31]. The structure of enzymes, human AChE (*hr*AChE, PDB ID: 1B41) [32], *Torpedo Californica* AChE (*TcAChE*, PDB ID: 2CKM) [33] and *Electric eel* AChE (*Ee*AChE, PDB ID: 1C2O) [34] were prepared using Pymol 1.3 from crystal structures [21]. Both the compounds and the enzymes were prepared for docking using the AutoDock Tools program (1.5.2). The reactivators were modelled as fully charged. Water molecules and other non-enzymatic molecules were removed (*i.e.*, withdrawing the fasciculin 2 from *hr*AChE, bis(7)-tacrine from *TcAChE*) and polar hydrogen atoms were added. The 3D affinity grid box in the *x*-, *y*- and *z*-axes were 66, 66 and 66 with spacing 0.253 Å for *hr*AChE, within the *TcAChE* grid box dimensions were set to *x* = 60, *y* = 64, *z* = 64 with spacing 0.253 Å and *x* = 30, *y* = 30, *z* = 30 with spacing 0.253 Å for *Ee*AChE. For the *hr*AChE docking, the grid for energy was set in the coordinates *x* = 119.775, *y* = 117.597 and *z* = −128.964, within *TcAChE* the coordinates were adjusted to *x* = −2.122, *y* = 60.902 and *z* = 61.812 and for *Ee*AChE those were *x* = 8.209, *y* = 65.726 and *z* = 63.335. In the *hr*AChE enzyme residues: Trp86, Tyr72, Trp286, Asp74, Tyr341, His447 and Phe297 were set to have flexible side chains. In the *TcAChE* enzyme, amino-acid residues: Tyr121, Ser200, Phe290, Phe331, Phe330, Tyr442, Trp84, His440, Phe288, Tyr130, Tyr334, Trp432 and Trp279 side chains were set as flexible, for *Ee*AChE those were Trp86, Tyr133, Trp439, Tyr449, Tyr337, Tyr341, Ser203, His447, Phe338, Phe295, Phe297, Tyr124 and Trp286. Flexible ligand docking was performed for the selected compound **13** with default settings. Docking for all **13**/AChE complexes was repeated 10 times. The docking calculations were performed on a Mac Pro 4.1 Quad-Core Intel Xeon 2.93 GHz and partially with the use of computer resources of Czech National Grid Infrastructure MetaCentrum. Visualization was performed in Pymol 1.3.

## 4. Conclusions

The main aim of this study was the determination of a structure-activity relationship for 24 oxime based compounds and their ability to inhibit *hr*AChE. In addition, we compared the inhibitory potency of these compounds as found in *hr*AChE to those previously determined in *Ee*AChE. Our results revealed that the important structural factors for the design and synthesis of novel peripherally acting *hr*AChE inhibitors are firstly, linker length (>5 carbons), and secondly, the positions of the oxime groups on the pyridinium rings (*ortho* to the pyridinium nitrogen revealed the highest inhibitory potency). Docking calculations justify the low IC_50_ values of **13** by predicting its binding pose that altered the conformations of residues in the catalytic triad while simultaneously occupying the mid-gorge and contacted PAS (peripheral active site) in both enzymes. We also showed that the *hr*AChE enzyme is generally more sensitive to inhibition by these compounds than the *Ee*AChE enzyme. Moreover, reversible inhibition needs to be taken into account when screening new, bis-pyridinium oxime based compounds for reactivation potential.

As these compounds contain quaternary ammonium moieties which limit permeability through the blood-brain barrier, they could be used for medical conditions as peripherally acting agents. Inhibitors of peripheral *hr*AChE are commonly used in the clinic for treatment of myasthenia gravis or in anesthetic practice to reverse the skeletal muscle relaxation induced by non-depolarizing neuromuscular blocking agents. Several of the compounds presented in this study could be used as new therapeutic leads for these peripheral indications.

## Figures and Tables

**Table 1:** Screening of IC_50_ values of mono-pyridinium reactivators as *Ee*AChE or *hr*AChE inhibitors with the oxime group at various positions on the pyridinium ring.

Compound	Name of oxime	Oxime position	IC_50_ values *Ee*AChE (mM)	IC_50_ values *hr*AChE (mM)	95% confidence intervals (mM)
**1**	2PAM	2	54.9	45.1	26.5–160.6
**2**	3PAM	3	27.1	41.6	8.96–192.8
**3**	4PAM	4	64.3	-	-

Three independent determinations were performed for each IC_50_. Confidence interval is related to *hr*AChE testing.

**Table 2:** Sreening of IC_50_ values of oximes as *hr*AChE inhibitors with the oxime group at various positions on the pyridinium ring or with various linker lengths between the pyridinium rings.

Compound	Name of oxime	*n*	Oxime position	IC_50_ values *Ee*AChE (mM)	IC_50_ values rec-*hr*AChE (mM)	95% confidence intervals (mM)
**4**	K154	1	4,4′	227.4	21.38	-
**5**	K191	2	4,4′	124.6	3.69	0.94–10.75
**6**	K018	3	4,4′	51.9	24.37	13.84–42.92
**7**	K074	4	4,4′	4.3	3.44	1.92–6.15
**8**	K305	5	4,4′	0.9	1.34	0.66–2.72
**9**	K194	6	4,4′	0.9	0.65	0.64–1.07
**10**	K309	7	4,4′	0.7	0.33	0.27–0.42
**11**	K197	8	4,4′	0.4	0.14	0.09–0.21
**12**	K310	9	4,4′	0.3	0.08	0.04–0.19
**13**	K338	10	4,4′	0.1	0.05	0.03–0.08
**14**	K339	11	4,4′	0.4	0.08	0.04–0.13
**15**	K340	12	4,4′	0.5	0.08	0.06–0.11
**16**	K005	3	2,2′	0.4	2.23	1.76–2.83
**17**	K099	3	3,3′	19.5	7.01	3.63–13.51
**18**	K207	3	2,3′	17.7	7.09	4.08–12.36
**19**	K208	3	2,4′	3.1	4.26	2.43–7.49
**20**	K209	3	3,4′	13.8	8.06	6.36–10.22
**21**	K033	4	2,2′	1.1	1.14	0.81–1.62
**22**	K101	4	3,3′	4.8	10.29	7.19–14.73

Three independent determinations were performed for each IC_50_. Confidence interval is related to *hr*AChE testing.

**Table 3:** Screening of IC_50_ values of oximes as *hr*AChE inhibitors with the oxime at various positions on the pyridinium ring or with various linker lengths between the pyridinium rings.

Compound	Name of oxime	*n*	Oxime position	IC_50_ values *Ee*AChE (mM)	IC_50_ values *hr*AChE (mM)	95% confidence intervals (mM)
**6**	K018	3	4,4′	51.9	24.37	13.84–42.92
**23**	K318	CC=CC	4,4′	36.7	17.33	8.22–36.52
**7**	K074	4	4,4′	4.3	3.44	1.92–6.15
**24**	K075	C-O-C	4,4′	6.7	8.09	4.51–9.91

Three independent determinations were performed for each IC_50_. Confidence interval is related to *hr*AChE testing.

**Table 4:** Residues of *Tc*AChE and *Ee*AChE matched in *R* = 8 Å spherical selections centered on His440/His447.

Enzyme	Amino acid residues														
***Tc*AChE**	GLY 80	MET 83	TRP 84	GLY 117	GLY 118	GLY 119	GLU 199	SER 200	ALA 201	GLN 225	SER 226	GLY 227	CYS 231	TRP 233	PHE 288
***Ee*AChE**	GLY 82	MET 85	TRP 86	GLY 120	GLY 121	GLY 122	GLU 202	SER 203	ALA 204	GLN 228	SER 229	GLY 230	THR 231	TRP 236	PHE 295
***Tc*AChE**	PHE 290	VAL 323	ASN 324	LYS 325	ASP 326	GLU 327	GLY 328	SER 329	PHE 330	PHE 331	GLY 396	ASN 399	VAL 400	TRP 432	MET 436
***Ee*AChE**	PHE 297	VAL 330	VAL 331	LYS 332	ASP 333	GLU 334	GLY 335	SER 336	TYR 337	PHE 338	GLY 403	ASN 406	VAL 407	TRP 439	MET 443
***Tc*AChE**	GLY 437	VAL 438	ILE 439	HIS 440	GLY 441	TYR 442	GLU 443	ILE 444	-						
***Ee*AChE**	GLY 444	VAL 445	PRO 446	HIS 447	GLY 448	TYR 449	GLU 450	ILE 451							
